# CX3CR1 Acts as a Protective Biomarker in the Tumor Microenvironment of Colorectal Cancer

**DOI:** 10.3389/fimmu.2021.758040

**Published:** 2022-01-24

**Authors:** Yuanyi Yue, Qiang Zhang, Zhengrong Sun

**Affiliations:** ^1^ Department of Gastroenterology Medicine, Shengjing Hospital of China Medical University, Shenyang, China; ^2^ Department of Pulmonary and Critical Care Medicine, Shengjing Hospital of China Medical University, Shenyang, China; ^3^ BioBank, Shengjing Hospital of China Medical University, Shenyang, China

**Keywords:** colorectal cancer, tumor microenvironment, ESTIMATE algorithm, stromal, immune, prognosis, CX3CR1

## Abstract

The tumor microenvironment (TME) plays an important role in the pathogenesis of many cancers. We aimed to screen the TME-related hub genes of colorectal adenoma (CRAD) and identify possible prognostic biomarkers. The gene expression profiles and clinical data of 464 CRAD patients in The Cancer Genome Atlas (TCGA) database were downloaded. The Estimation of STromal and Immune cells in MAlignant Tumours using Expression data (ESTIMATE) algorithm was performed to calculate the ImmuneScore, StromalScore, and EstimateScore. Thereafter, differentially expressed genes (DEGs) were screened. Gene Ontology (GO), Kyoto Encyclopedia of Genes and Genomes (KEGG) pathway, and protein–protein interaction (PPI) analysis were performed to explore the roles of DEGs. Furthermore, univariate and multivariate Cox analyses were accomplished to identify independent prognostic factors of CRAD. CX3CR1 was selected as a hub gene, and the expression was confirmed in colorectal cancer (CRC) patients and cell lines. The correlations between CX3CR1 and tumor-infiltrating immune cells were estimated by Tumor IMmune Estimation Resource database (TIMER) and CIBERSORT analysis. Besides, we investigated the effects of coculture with THP-1-derived macrophages with HCT8 cells with low CX3CR1 expression on immune marker expression, cell viability, and migration. There were significant differences in the ImmuneScore and EstimateScore among different stages. Patients with low scores presented significantly lower lifetimes than those in the high-score group. Moreover, we recognized 1,578 intersection genes in ImmuneScore and StromalScore, and these genes were mainly enriched in numerous immune-related biological processes. CX3CR1 was found to be associated with immune cell infiltration levels, immune marker expression, and macrophage polarization. Simultaneous silencing of CX3CR1 and coculture with THP-1 cells further regulated macrophage polarization and promoted the cell proliferation and migration of CRC cells. CX3CR1 was decreased in CRAD tissues and cell lines and was related to T and N stages, tumor differentiation, and prognosis. Our results suggest that CX3CR1 contributes to the recruitment and regulation of immune-infiltrating cells and macrophage polarization in CRC and TAM-induced CRC progression. CX3CR1 may act as a prognostic biomarker in CRC.

## Introduction

Colorectal cancer (CRC) is one of the most common malignant gastrointestinal cancers (1) and ranks the fifth leading cause of cancer-related death in China (2). CRC has been well-acknowledged as a heterogeneous disease, which presents various differences in clinical features, molecular genetic alterations, and prognosis (3). Some factors, such as age, diet, environment, unhealthy lifestyle, obesity, inflammatory bowel disease (IBD), gene mutation, gut microbiota, and family history of colon cancers, have been reported to be at high risk of developing the tumor (4–7). Among the influencing factors, molecular genetic changes have been considered as one of the important key characteristics contributing to the progression of cancers (8, 9).

Growing evidence suggests that the tumor microenvironment (TME) plays a critical role in the progression and prognosis of malignant tumors (10, 11), including CRC (12, 13). The TME is the location of tumor appearance, comprising many cells, mediators, and molecules (14, 15). Among the cells, infiltrating stromal and immune cells are the two foremost members of the TME, which significantly contributes to cancer biology (16, 17). It has been demonstrated that the early stage of CRC is characterized by a high content of stromal cells and the infiltration of immune cells, with unfavorable and favorable prognosis of refeeding syndrome (RFS), respectively (18). In addition, the immune and stromal stratification of CRC is responsible for molecular subtypes and tailored immunotherapy (19). However, little information is available regarding the TME-related genes that could identify potential prognostic biomarkers for CRC. The Estimation of STromal and Immune cells in MAlignant Tumours using Expression data (ESTIMATE) algorithm is an accurate method to calculate the specific gene data expression signature to evaluate the infiltration of stromal and immune cells and tumor purity. It is a broad, novel, and reliable algorithm that has been administered in data mining of several cancers, and this method has been proven effective in several large independent databases (20–24). ESTIMATE algorithm includes Immunescore, StromalScore, and ESTIMATEScore. Immunescore is the percentage of Immune cells, which is a scoring system based on the quantitative analysis of cytotoxic T cells and memory T cells in the core of the tumor (CT) and the invasive margin (IM) of the tumor (25). StromalScore is the percentage of stromal cells, and EstimateScore is the sum of the ImmuneScore and StromalScore (26). A higher ImmuneScore or StromalScore is indicative of the presence of a significant immune or stromal component in the TME, and ESTIMATEScore is the sum of immune and stromal score. In other words, high tumor purity is related to the unfavorable prognosis of patients. Although the ESTIMATE algorithm is based on cancer tissue data, it is effective in evaluating cellular data as well (27). Several studies have confirmed that the scores are associated with the clinicopathological characteristics and chemotherapeutic drug resistance in various types of tumors, and that ESTIMATE could be used as an indicator for patient prognosis assessment (27–29).

Therefore, in the current study, we aimed to evaluate the ImmuneScore and StromalScore in the TME based on CRC data acquired from The Cancer Genome Atlas (TCGA) database by single-sample gene set enrichment analysis (ssGSEA). Moreover, we further explored the stromal-immune score-based gene signature related to the prognosis of CRC. Our results might shed insight into the improvement of novel prognostic biomarkers and treatments, specifically immunotherapies, for patients with CRC.

## Materials and Methods

### The Cancer Genome Atlas Data Download and Processing

In this analysis, we downloaded the expression datasets of fragments per kilobase of exon per million mapped fragments (FPKM) from TCGA website (https://portal.gdc.cancer.gov/). The samples were screened according to the clinical information. The principles of sample selection were listed as follows: 1) Primary tumor tissues were selected; 2) Complete information for tumor-node-metastasis (TNM) staging and stage were selected, and the samples without relevant follow-up data and incomplete information were removed; 3) The samples without clinical survival information were removed; 4) Five-year survival data could be obtained from the patients whose survival time is more than 1 month and less than 5 years. According to the above inclusion and exclusion criteria, a total of 464 colorectal adenoma (CRAD) samples were included for subsequent analysis.

### Calculation of ImmuneScore, StromalScore, and EstimateScore

After selecting the samples, we extracted the expression matrix from the samples and then calculated the immune purity of the expression matrix using the “estimate” R package. We performed ssGSEA method for each sample, and the immune infiltration (ImmuneScore), overall stromal content (StromalScore), and the combined (EstimateScore) were calculated by ESTIMATE algorithms (21).

### Overall Survival Analysis

Kaplan–Meier plots were performed to investigate the prognosis of patients with CRAD. The individuals were assigned to the high-score group (the values of >optimal cutoff) and low-score group (the values of <optimal cutoff) based on the optimal cutoff values of the ImmuneScore and StromalScore. Maximally selected rank statistics (30) were performed to ascertain the optimal cutoff. The “survminer” in R package was performed to detect the survival analyses.

### Selection of Differentially Expressed Genes

The patients were divided into high-score and low-score groups based on the optimal cutoff mentioned above. The selection of DEGs was performed according to the published method (31) by using “edgeR” R package with *P*-value <0.01 and |logFC| >1. Volcano plot was further used to visualize the DEGs. Moreover, Venn diagrams were performed to detect the upregulated or downregulated intersection genes of DEGs in the immune and stromal groups using a website tool (http://bioinformatics.psb.ugent.be/webtools/Venn/).

### Enrichment Analysis of Intersection Genes

Gene Ontology (GO) and Kyoto Encyclopedia of Genes and Genomes (KEGG) enrichment analyses were performed by “ClusterProfiler” R package and ClueGO plug-in in Cytoscape software (3.6.1 version) (32).

### Construction of Protein–Protein Interaction Network

The PPI network was constructed by STRING (http://string-db.org) (33) with an interaction combined score >0.7. The interaction nodes of the protein were visualized by using Cytoscape (34), and enrichment analysis of each cluster was analyzed with ClueGo software (35). In addition, Molecular Complex Detection (MCODE) was used to investigate the key subnetworks in PPI networks. The parameters of clustering and scoring were selected as follows: MCODE score ≥5, degree cutoff = 2, node score cutoff = 0.2, max depth = 100, and k-score = 2. Genes with the highest MCODE score in the PPI network were selected as the hub genes.

### Univariate and Multivariate Cox Analyses

Univariate and multivariate Cox analyses were performed to assess the independent prognostic factors associated with patients’ survival. Hub gene expression, T stage, N stage, M stage, and Stage were selected as covariates. Hazard ratios (HRs) were used to recognize protective (HR <1) or risky genes (HR >1), and the most relevant gene for the prognosis of CRAD was obtained by regression analysis.

### Tumor Microenvironment Analysis

The abundance of immune infiltrates was estimated by Tumor IMmune Estimation Resource database (TIMER; cistrome.shinyapps.io/timer) (36) and CIBERSORT analysis. The correlation between CX3CR1 expression and the abundance of infiltrating immune cells, including tumor purity, B cells, CD8^+^ T cells, CD4^+^ T cells, macrophages, neutrophils, and dendritic cells (DCs), was analyzed. Furthermore, the interconnections between CX3CR1 expression and molecular biomarkers of tumor-infiltrating immune cells were investigated by correlation modules. For further investigation, CIBERSORT was used to estimate the abundance of different immune cell types in the TME. It is a deconvolution algorithm for calculating the abundance of immune cell infiltration for each sample, which is based on a gene set of 22 sets of immune cell-associated genes (37) (https://cibersort.stanford.edu/). RNA sequencing (RNA-seq) data of CRC samples were divided into low CX3CR1 expression group and high CX3CR1 expression group according to the median level of CX3CR1. Data were imported into CIBERSORT and LM22 signature matrix.

### Subjects

A total of 60 (38 males and 22 females, mean age: 58 years old) CRC tumors and matched adjacent non-tumor tissues were acquired from subjects at Shengjing Hospital of China Medical University between 2014 and 2015. The collected samples were immediately frozen after the operation and stored at -80°C until use. Patients’ information, including gender, ages, tumor location, size, TNM classification, and differentiation, was collected. All individuals did not receive any preoperative treatments. Our study was permitted by the Medical Ethics Committee of Shengjing Hospital of China Medical University (No. 2014PS13), and informed consent was acquired from each individual.

### Cell Lines

Human normal intestinal mucous cell line CCC-HIE-2, human CRC cell lines (CaCO-2, HCT8, HCT-116, and LoVo), and human THP-1 monocytes were acquired from the American Type Culture Collection (ATCC, Manassas, VA, USA). These cell lines were routinely cultured in Dulbecco’s modified Eagle’s medium (DMEM; Gibco, Grand Island, NY) supplemented with 10% fetal bovine serum (FBS; Gibco) and 2 mM L-glutamine (Gibco), 100 U/ml penicillin (Invitrogen, Carlsbad, CA, USA), and 100 µg/ml streptomycin (Invitrogen). They were maintained at 37°C in a 5% CO_2_ atmosphere.

### Coculture of THP-1 With HCT8

The CRC cells HCT8 and THP-1-derived macrophages were cocultured with a non-contact cell culture insert (0.4 μM; Corning, NY, USA). The THP-1 cells were seeded into the upper chamber at a density of 5 × 10^5^ cells/ml, and they were induced to differentiate into M2 macrophages by administration of 350 nm phorbol-12-myristate-13-acetate (PMA; Sigma-Aldrich, St. Louis, MO, USA) for 6 h and interleukin (IL)-4 for 18 h. The ratio of M2 cells to HCT8 cells was 1:4. After washing with phosphate-buffered saline (PBS), the cells were incubated for another 24 h to remove the effect of PMA. The HCT8 cells (2.5 × 10^5^ cells/ml) were placed in the lower chamber for 24 h to allow adherence. Thereafter, the THP-1-derived macrophages were directly put on the top of plates containing the HCT8 cells and were then incubated for 24 h in serum-free RPMI 1640.

### Transient Transfection

Small interfering RNA for CX3CR1 (si-CX3CR1, 150 nM) was transfected into HCT8 to knockdown CX3CR1. si-CX3CR1 was designed and produced by Genechem (Shanghai, China). The sequence of si-CX3CR1 was as follows: 5′-CTTGTCTGATCTGCTGTTT-3′. Cell transfection was performed by using Lipofectamine^®^ 2000 (Invitrogen) at indicated times.

### Cell Counting Kit-8

After transfection with si-NC or si-CX3XR1 in the absence or presence of coculture, the HCT8 cells were seeded into 96-well plates (5 × 10^3^ cells/well). Thereafter, the cell viability was assessed using Cell Counting Kit-8 (CCK-8; Japan Dojindo Molecular Technologies) at 24, 48, 72, and 96 h according to the manufacturer’s instructions.

### Migration Assay

After transfection with si-NC or si-CX3XR1 in the absence or presence of coculture, cell migration assay was conducted using 24-well Transwell plates (8.0 μm; Corning, NY, USA). The macrophages or cancer cells (5 × 10^4^, HCT8-si-NC, HCT-8-si-CX3CR1) were planted into the upper chambers, and 600 µl RPMI 1640 containing 10% FBS were placed into the lower chambers. Thereafter, the Transwell plates were incubated in a 37°C, 5% CO_2_ incubator for 48 h and then fixed in 4% formaldehyde for half an hour and stained with 0.01% crystal violet. Non-migrating cells were carefully removed with a cotton swab, and the cells that had migrated to the lower chambers were counted under the microscope.

### Quantitative Real-Time Reverse Transcription PCR

Total RNA was extracted from the samples, and cells using TRIzol reagent (Invitrogen). Reverse-transcription reactions were performed using an M-MLV Reverse Transcriptase kit (Roche Molecular Biochemicals) according to the manufacturer’s protocol. Real-time PCR was carried out using a standard SYBR Green PCR kit (Qiagen, Hilden, Germany). Glyceraldehyde-3-phosphate dehydrogenase (GAPDH) was used as an internal reference. The sequences of different primers were summarized in **Table 1**. Each sample was analyzed in triplicate, and relative quantitation of gene expression levels was determined using 2^-△△CT^ method.

**Table 1:** The sequences of different primers.

Gene	Sequence (5′ -> 3′)	
*CX3CR1*	Forward	ACTTTGAGTACGATGATTTGGCT
	Reverse	GGTAAATGTCGGTGACACTCTT
*NOS2*	Forward	TTCAGTATCACAACCTCAGCAAG
	Reverse	TGGACCTGCAAGTTAAAATCCC
*IRF5*	Forward	GGGCTTCAATGGGTCAACG
	Reverse	GCCTTCGGTGTATTTCCCTG
*PTGS2*	Forward	CTGGCGCTCAGCCATACAG
	Reverse	CGCACTTATACTGGTCAAATCCC
*CD163*	Forward	TTTGTCAACTTGAGTCCCTTCAC
	Reverse	TCCCGCTACACTTGTTTTCAC
*VSIG4*	Forward	GGGGCACCTAACAGTGGAC
	Reverse	GTCTGAGCCACGTTGTACCAG
*MS4A4A*	Forward	ACCATGCAAGGAATGGAACAG
	Reverse	TTCCCATGCTAAGGCTCATCA
*GAPDH*	Forward	ACACCCACTCCTCCACCTTT
	Reverse	TTACTCCTTGGAGGCCATGT

CX3CR1, C-X3-C motif chemokine receptor 1; NOS2, nitric oxide synthase 2; IRF5, interferon regulatory factor 5; PTGS2, prostaglandin-endoperoxide synthase 2; VSIG4, V-set and immunoglobulin domain containing 4; membrane-spanning 4-domains, subfamily A, member 4A; GAPDH, glyceraldehyde-3-phosphate dehydrogenase.

### Western Blot

Proteins were extracted from the samples and cells using radioimmunoprecipitation assay (RIPA) lysis buffer (Beyotime, Shanghai, China). Thereafter, the acquired proteins were separated using sodium dodecyl sulfate (SDS)-polyacrylamide gel electrophoresis (PAGE) and transferred onto polyvinylidene fluoride (PVDF) membranes (Beyotime). The membranes were then incubated with anti-CX3CR1 primary antibody (SAB2900202; Sigma-Aldrich, St. Louis, MO, USA) at 4°C overnight and with horseradish peroxidase-conjugated secondary antibody (A2691, Sigma-Aldrich) for 1 h at room temperature. Enhanced chemiluminescence (ECL) plus Kit (GE Healthcare, Little Chalfont, Buckinghamshire, UK) was used to analyze the chemiluminescence intensity of each membrane and then quantitated by ImageJ software (NIH, Bethesda, MD, USA). GAPDH was used as a housekeeping gene.

### Statistical Analyses

All analyses were conducted with R version 3.5.3 (http://www.R-project.org), along with its appropriate packages. Survival analysis was performed using Kaplan–Meier method with the log-rank test. Univariate and multivariate analysis Cox proportional hazards model was used to assess the potential independent factors with the prognosis. For the *in vitro* experiments, the acquired data are presented as the mean  ± standard deviation (SD). The differences were evaluated with Student’s *t*-tests (for 2 groups) or one-way analysis of variance (ANOVA) for 3 and/or more than 3 groups using SPSS Statistics 19.0 software (IBM, Armonk, NY, USA). For the CIBERSORT algorithm, it was performed with 1,000 simulations, and the results were filtered according to *P* < 0.05. After obtaining the abundance of immune cell infiltration in each sample, correlations between these immune cells and CX3CR1 expression levels were calculated based on the Spearman coefficient, and differences in immune cell infiltration between high and low CX3CR1 expression groups were calculated using the Wilcoxon log-rank test. *P* < 0.05 was regarded as statistically significant.

## Results

### ImmuneScore and ESTIMATEScore Correlate With Clinical Data and Prognosis in Patients with Colorectal Adenoma

**Figure 1:**
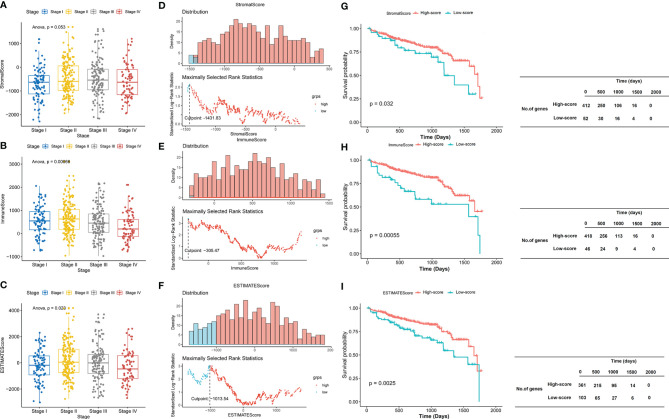
ImmuneScore and ESTIMATEScore correlate with clinical data and prognosis in patients with CRAD. **(A–C)** The boxplot of StromalScore, ImmuneScore, and ESTIMATEScore of CRAD patients in different stages. **(D–F)** The distribution of StromalScore, ImmuneScore, and ESTIMATEScore. **(G–I)** Kaplan–Meier survival analysis of StromalScore, ImmuneScore, and ESTIMATEScore. CRAD, colorectal adenoma.

A total of 464 samples were used to analyze in the current study according to TCGA data. ESTIMATE algorithm was used to calculate the StromalScore, ImmuneScore, and ESTIMATEScore. According to the clinical data extracted from TCGA (**Supplementary Table S1**), we observed that there was no significant difference among different stages in the StromalScore (*P* = 0.053; **Figure 1A**). However, there were statistical differences among different stages in the ImmuneScore (*P* = 0.00066; **Figure 1B**) and ESTIMATEScore (*P* = 0.023; **Figure 1C**). The StromalScore ranged from -2,286.02 to 1,695.44, ImmuneScore ranged from -741.19 to 2,489.81, and ESTIMATEScore ranged from -3,027.21 to 4,185.25. The scores were summarized in **Supplementary Table S2**. The distribution of StromalScore, ImmuneScore, and ESTIMATEScore was shown in **Figures 1D–F**, and the cut points respectively were -1,431.83, -305.47, and -1,013.54. To further explore the potential correlation between clinical overall survival (OS) of patients with CRAD and their three scores, we assigned the 464 patients into the high-score group (the values >optimal cutoff) and the low-score group (the values <optimal cutoff). Thereafter, we assessed the potential correlation with Kaplan–Meier survival analysis. The results showed that patients with high scores presented significantly longer lifetimes than those in the low-score group for StromalScore (*P* = 0.032; **Figure 1G**), ImmuneScore (*P* = 0.00055; **Figure 1H**), and ESTIMATEScore (*P* = 0.0025; **Figure 1I**). These results implied that both ImmuneScore and ESTIMATEScore correlated with clinical data and prognosis in patients with CRAD, while StromalScore only correlated with the prognosis but not the clinical data.

### Identification of Differentially Expressed Genes Based on ImmuneScore and StromalScore

**Figure 2:**
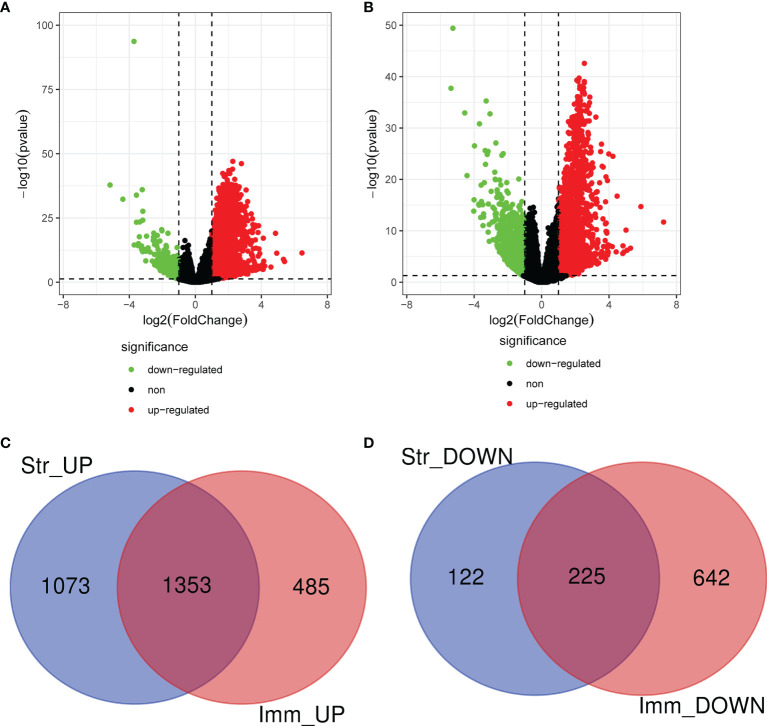
Identification of DEGs based on ImmuneScore and StromalScore. **(A)** The distribution of DEGs of ImmuneScore using volcano plots. **(B)** The distribution of DEGs of StromalScore using volcano plots. **(C)** Upregulated intersection genes of DEGs detected by Venn diagrams. **(D)** Downregulated intersection genes of DEGs detected by Venn diagrams. DEGs, differentially expressed genes.

The expression profile data of 464 patients with CRAD were further examined to detect DEGs by using “edgeR” R package. A total of 2,773 and 2,705 DEGs were respectively screened in CRAD sample cells based on ImmuneScore and StromalScore. Volcano plots were performed to visualize the distribution of DEGs of ImmuneScore and StromalScore. Upregulated or downregulated genes were characterized respectively with red or green dots (**Figures 2A, B** **)**. Venn diagrams were accomplished to detect the upregulated or downregulated intersection genes of DEGs. Among them, we recognized 2,426 upregulated genes and 347 downregulated genes in StromalScore and 1,838 upregulated genes and 867 downregulated genes in ImmuneScore. A total of 1,353 upregulated intersection genes and 225 downregulated intersection genes were selected for further analysis (**Figures 2C, D** **)**. Upregulated and downregulated DEGs were respectively listed in **Supplementary Tables S3**, **S4**.

### Gene Ontology and Kyoto Encyclopedia of Genes and Genomes Pathway Enrichment Analyses

**Figure 3:**
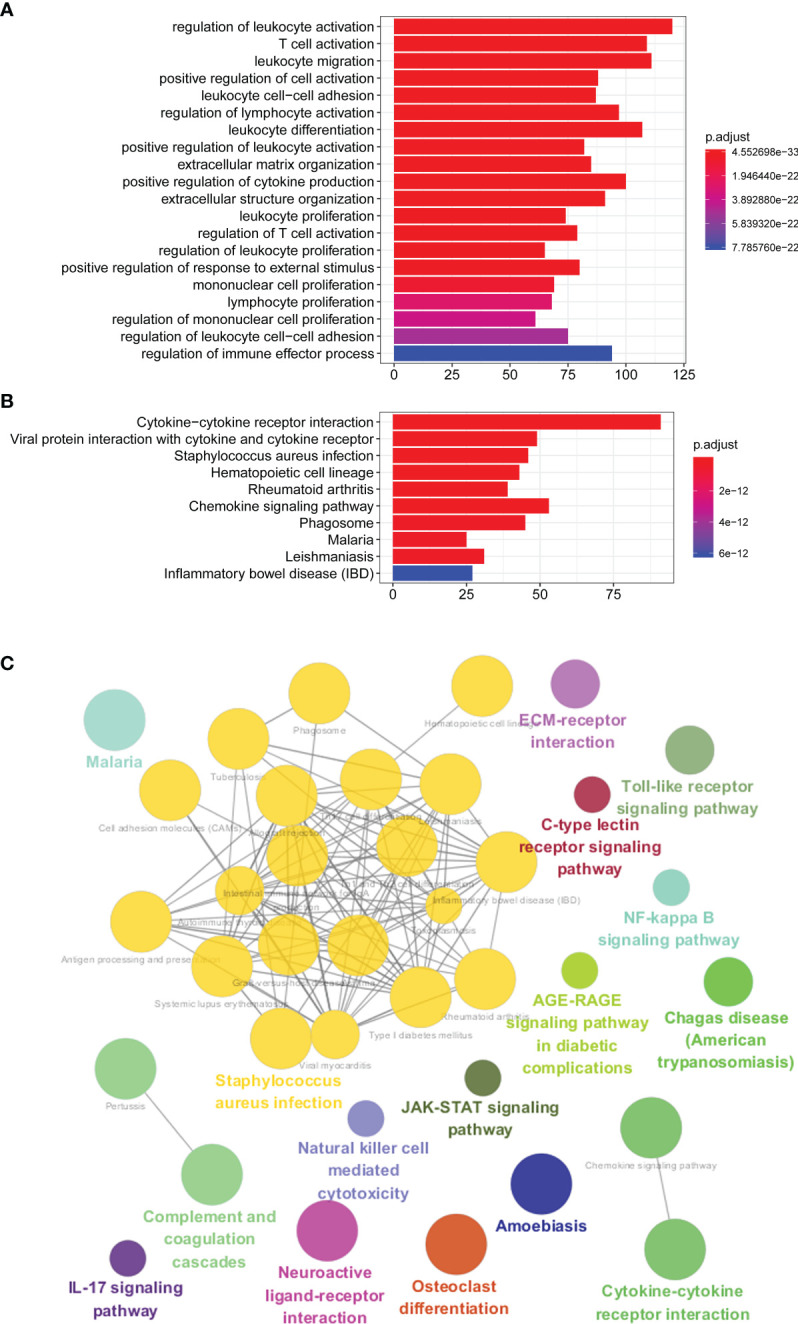
GO and KEGG pathway enrichment analyses. **(A)** Top 20 GO terms of the intersection DEGs using “ClusterProfiler” R package. **(B)** Top 10 KEGG pathways of the intersection DEGs using “ClusterProfiler” R package. **(C)** GO terms and KEGG pathway using ClueGO method. GO, Gene Ontology; KEGG, Kyoto Encyclopedia of Genes and Genomes; DEGs, differentially expressed genes.

**Figure 4:**
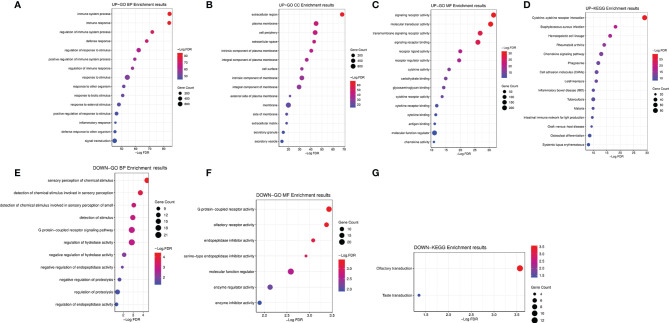
The dotplot for the enriched GO and KEGG analysis of upregulated and downregulated DEGs. **(A)** The dotplot for the enriched GO BP of upregulated DEGs. **(B)** The dotplot for the enriched GO CC of upregulated DEGs. **(C)** The dotplot for the enriched GO MF of upregulated DEGs. **(D)** The dotplot for the enriched KEGG analysis of upregulated DEGs. **(E)** The dotplot for the enriched GO BP of downregulated DEGs. **(F)** The dotplot for the enriched GO MF of downregulated DEGs. **(G)** The dotplot for the enriched KEGG analysis of downregulated DEGs. GO, Gene Ontology; KEGG, Kyoto Encyclopedia of Genes and Genomes; DEGs, differentially expressed genes; BP, biological process; MF, molecular function; CC, cellular component.

We further explored the GO and KEGG pathway enrichment analysis of 1,578 intersection genes by two different methods: the “ClusterProfiler” R package and the ClueGO plug-in in Cytoscape software. All the GO terms and KEGG pathways were recorded in **Supplementary Tables S5**, **S6**, respectively. Top 20 GO terms and Top 10 KEGG pathways were presented in the current study using the “ClusterProfiler” R package. As shown in **Figure 3A**, we found that the DEGs were mainly enriched in the regulation of lymphocyte activation, T-cell activation, leukocyte migration, positive regulation of cell activation and leukocyte cell–cell adhesion, and so on. Moreover, the KEGG enrichment analysis of DEGs was primarily enriched in cytokine–cytokine receptor interaction, viral protein interaction with cytokine and cytokine receptor, *Staphylococcus aureus* infection, hematopoietic cell lineage, rheumatoid arthritis, and chemokine signaling pathway, etc. (**Figure 3B**). The results of GO terms and KEGG pathway using ClueGO method were shown in **Figure 3C**. Interestingly, we found that the results of immune-related genes in GO term biological process (BP) and KEGG pathways were achieved only from the upregulated DEGs. The dotplot for the enriched GO and KEGG analysis of upregulated and downregulated DEGs was demonstrated in **Figure 4**. Therefore, we performed further analyses on upregulated DEGs only.

### Protein–Protein Interaction Construction and Module Analysis of Upregulated Differentially Expressed Genes

**Figure 5:**
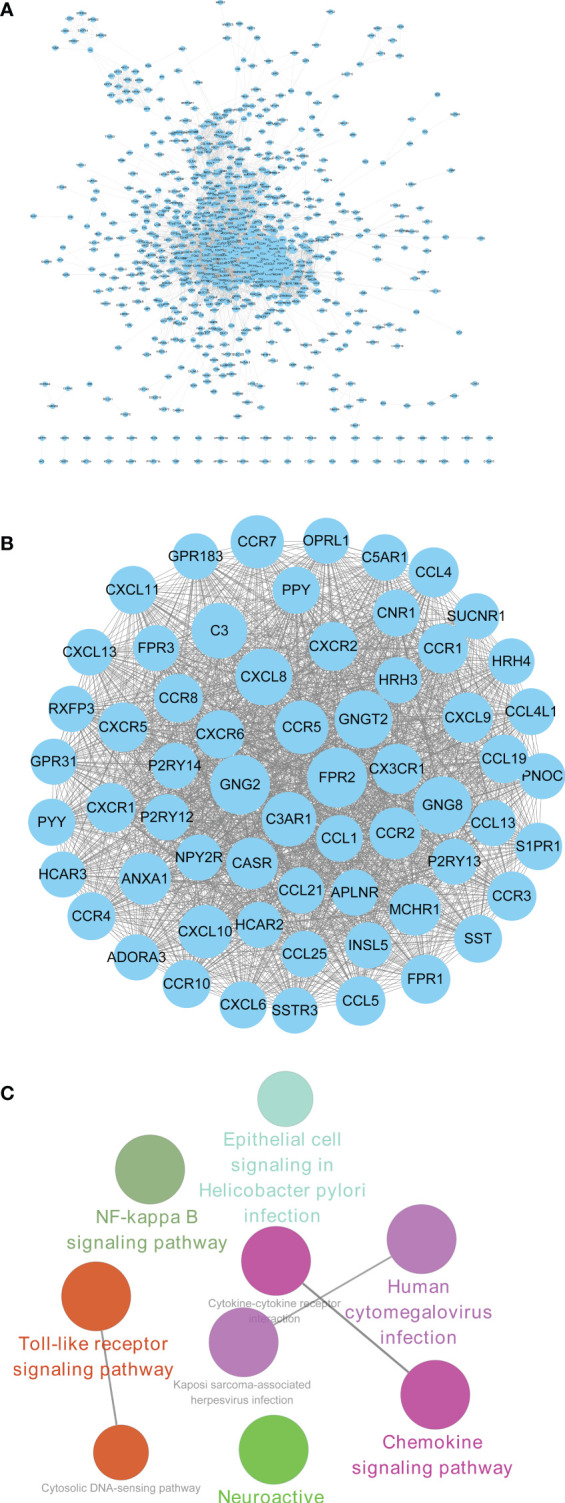
PPI construction and module analysis of upregulated DEGs. **(A)** PPI network of upregulated intersection DEGs using STRING tool and Cytoscape software. **(B)** The first significant module with 62 hub genes. **(C)** The functional analysis of 62 hub genes screened by the Cytoscape software with ClueGO plug-in. PPI, protein–protein interaction; DEGs, differentially expressed genes.

PPI network of upregulated 1,353 DEGs for CRAD was constructed with STRING tool and Cytoscape software (**Figure 5A**). There were 836 nodes in the network with the interaction combined score >0.7 and 6,662 pairs of interaction relationships (**Supplementary Table S7**). The circle size in the figure indicated the degree of the corresponding node. The larger the circle, the greater importance of the corresponding node in the network found. Furthermore, we used Cytotype MCODE software to investigate Clustering analysis of the above PPI network. According to the threshold value, we selected the first significant module with 62 hub genes (**Figure 5B** and **Supplementary Table S8** **)**. The functional analysis of 62 hub genes was preliminarily screened by the Cytoscape software with ClueGO plug-in. The results were shown in **Figure 5C**, which was consistent with the above KEGG results. Therefore, we confirmed that the analysis results were reliable.

### CX3CR1 Acts as a Biomarker of Progression and Prognosis in Colorectal Adenoma

**Figure 6:**
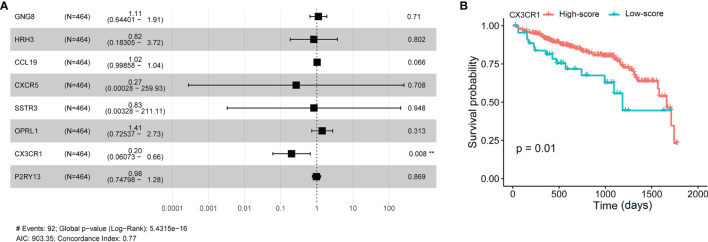
Multivariate Cox analyses of hub genes and survival analysis of CX3CR1 in patients with CRAD. **(A)** Multivariate Cox analyses of hub genes in patients with CRAD. **(B)** Survival analysis of CX3CR1 in patients with CRAD. GNG8, G protein subunit gamma 8; HRH3, histamine receptor H3; CRCL19, C-C motif chemokine ligand 19; CXCR5, C-X-C motif chemokine receptor 5; SSTR3, somatostatin receptor 3; OPRL1, opioid-related nociceptin receptor 1; CX3CR1, C-X3-C motif chemokine receptor 1; P2RY13, purinergic receptor P2Y13; CRAD, colorectal adenoma.

To further confirm the independent prognosis factors of patients with CRAD, we used iterative univariable Cox regression to judge the prognostic value of each gene included in the study. Then, we included all genes to conduct multivariable Cox regression, which employed Akaike information criterion (AIC)-based stepwise methods to train a model and is totally different from step 1. And genes that meet *P* < 0.05 of both univariable and multivariable Cox regression were deeded the prognostic genes. Finally, the number of these prognostic genes was eight. The univariate Cox analysis results were shown in **Table 2**, and we observed that there were significant differences in G protein subunit gamma 8 (GNG8), histamine receptor H3 (HRH3), C-C motif chemokine ligand 19 (CRCL19), C-X-C motif chemokine receptor 5 (CXCR5), somatostatin receptor 3 (SSTR3), opioid-related nociceptin receptor 1 (OPRL1), C-X3-C motif chemokine receptor 1 (CX3CR1), and purinergic receptor P2Y13 (P2RY13). The multivariate Cox analysis data showed that there was statistical significance in CX3CR1 (**Figure 6A**). All univariate and multivariate results were shown in **Supplementary Tables S9**, **S10**, respectively. We verified this result by analyzing the relationship between the expression of CX3CR1 and the prognosis of patients with CRAD. As indicated in **Figure 6B**, patients in the low-score group presented poorer 5-year survival consequences than those in the high-score group (*P* = 0.01). These data implied that CX3CR1 might act as a biomarker of progression and prognosis in patients with CRAD.

**Table 2:** Univariate Cox proportional hazards regression analyses of clinical parameters and hub genes in patients with CRAD.

Cells	coef	HR (95% CI for HR)	*P* value
GNG8	0.194	1.21 (1.11–1.32)	0.0000117
HRH3	0.433	1.54 (1.26–1.88)	0.0000183
CCL19	0.0167	1.02 (1.01–1.03)	0.000826
CXCR5	3.58	36 (3.6–359)	0.00228
SSTR3	1.61	5.01 (1.54–16.3)	0.00733
OPRL1	0.518	1.68 (1.08–2.6)	0.0202
CX3CR1	-1	0.367 (0.144–0.938)	0.0363
P2RY13	-0.259	0.772 (0.596–0.998)	0.0486

CI, confidence interval; HR, hazard ratio; GNG8, G protein subunit gamma 8; HRH3, histamine receptor H3; CRCL19, C-C motif chemokine ligand 19; CXCR5, C-X-C motif chemokine receptor 5; SSTR3, somatostatin receptor 3; OPRL1, opioid-related nociceptin receptor 1; CX3CR1, C-X3-C motif chemokine receptor 1; P2RY13, purinergic receptor P2Y13; CRAD, colorectal adenoma.

### CX3CR1 Is Associated With Immune Cell Infiltration Levels

**Figure 7:**
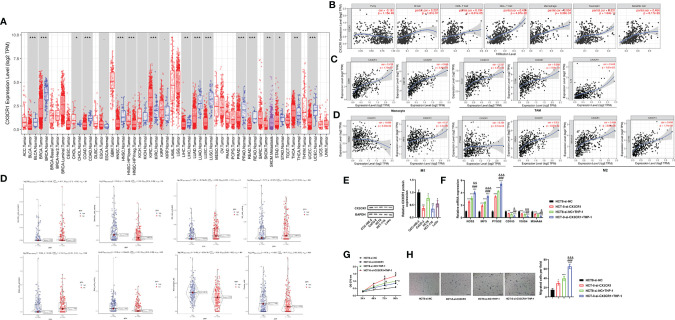
CX3CR1 is associated with immune cell infiltration levels and contributes to TAM-induced CRC progression. **(A)** The differential expression of CX3CR1 between tumor and adjacent normal tissues in COAD analyzed by the DiffExp module of the TIMER database. **(B)** The association between CX3CR1 expression and infiltration levels in COAD analyzed by the gene module of the TIMER database. **(C)** The correlation between CX3CR1 expression and immunological markers in COAD analyzed by the correlation module of the TIMER database. **(D)** The correlation between CX3CR1 expression and immune cell infiltration levels analyzed by CIBERSORT analysis. **(E)** The protein expression of CX3CR1 in different CRC cell lines. **(F–H)** The effects of coculture of THP-1 and HCT8 cells with low CX3CR1 expression on the mRNA expression of monocyte, TAM, M1 and M2 macrophage markers, proliferation and migration. *P < 0.05, ***P < 0.01, ***P < 0.001 compared to the HCT-8-si-NC group; ^#^ *P* < 0.05, ^##^ *P* < 0.01, ^###^ *P* < 0.001 compared to the HCT-8-si-CX3CR1 group; ^&^ *P* < 0.05, ^&&^ *P* < 0.01, ^&&&^ *P* < 0.001 compared to the HCT-8-si-NC+THP-1 group. CX3CR1, C-X3-C motif chemokine receptor 1; CRC, colorectal cancer; TAM, tumor-associated macrophage; COAD, colon adenocarcinoma; NOS2, nitric oxide synthase 2; IRF5, interferon regulatory factor 5; PTGS2, prostaglandin-endoperoxide synthase 2; VSIG4, V-set and immunoglobulin domain containing 4; membrane-spanning 4-domains, subfamily A, member 4A; TIMER, Tumor IMmune Estimation Resource database.

The differential expression of CX3CR1 between tumor and adjacent normal tissues was analyzed using the DiffExp module of the TIMER database. As demonstrated in **Figure 7A**, the results revealed that the levels of CX3CR1 were differentially expressed in various cancer types, including colon adenocarcinoma (COAD) (*P* < 0.001). It has been reported that tumor−infiltrating lymphocytes (TILs) are critical survival predictors in cancer patients, and tumor purity plays a significant role in determining CRC prognosis (38). Thus, we used the gene module of the TIMER database to explore whether CX3CR1 expression was related to infiltration levels in CRC. As shown in **Figure 7B**, CX3CR1 was negatively correlated with purity (cor = -0.161, *P* = 1.15e-03) and positively correlated with B cells (cor = 0.257, *P* = 1.61e-07), CD8^+^ T cells (cor = 0.194, *P* = 8.01e-07), CD4^+^ T cells (cor = 0.456, *P* = 4.97e-22), macrophages (cor = 0.534, *P* = 3.39e-31), neutrophils (cor = 0.331, *P* = 1.04e-11), and dendritic cells (DCs) (cor = 0.464, *P* = 8.17e-23). Furthermore, we examined the correlation between CX3CR1 expression and immune markers of different immune cells using the correlation module of the TIMER database in COAD, including monocyte markers (CD86 and CSF1R), tumor-associated macrophage (TAM) markers (CCL2, CD68, and IL10), M1 macrophage markers (NOS2, IRF5, and PTGS2), and M2 macrophage markers (VSIG4, MS4A4A, and CD163). The results showed that CX3CR1 expression was correlated with that of most monocytes, TAM, and M1 and M2 macrophage markers in COAD (**Figure 7C** **)**. For further exploration, CIBERSORT analysis indicated that the high expression of CX3CR1 was positively correlated with resting DCs (cor = 0.25, *P* = 1.75e-06), resting mast cells (cor = 0.21, *P* = 2.62e-05), M2 macrophages (cor = 0.33, *P* = 6.74e-10), and plasma cells (cor = 0.17, *P* = 0.002) and negatively correlated with activated DCs (cor = -0.11, *P* = 0.008), activated natural killer (NK) cells (cor = -0.20, *P* = 8.73e-05), and activated mast cells (cor = -0.14, *P* = 0.006). No significant difference was observed in resting NK cells (cor = -0.08, *P* = 0.123) (**Figure 7D**). These data suggested that CX3CR1 was associated with immune cell infiltration levels in CRC pathology.

### Coculture of THP-1 and HCT8 Cells With Low CX3CR1 Expression Regulates Macrophage Polarization and Promotes Proliferation and Migration

To further confirm the above results, we subsequently assessed the effects of coculture of THP-1 and CRC cells with low CX3CR1 expression on CRC cell functions. Firstly, we analyzed the protein expression of CX3CR1 in different CRC cell lines. The data revealed that the protein expression of CX3CR1 was significantly diminished in different CRC cell lines compared to the human normal intestinal mucous cell line CCC-HIE-2 (*P* < 0.05 or *P* < 0.01; **Figure 7E**), with the highest level in HCT8 cells. Then, the effects of coculture on the mRNA expression of M1 and M2 macrophage markers were explored. The findings revealed that, compared to the control group, silencing of CX3CR1 or coculture with THP-1 cells could significantly increase the mRNA levels of M1 macrophage markers (NOS2, IRF5, and PTGS2) (*P* < 0.001) but decrease the mRNA levels of M2 macrophage markers (VSIG4, MS4A4A, and CD163) (*P* < 0.01 or *P* < 0.001), while simultaneous silencing of CX3CR1 and coculture with THP-1 cells further enhanced the above functions (*P* < 0.05, *P* < 0.01, or *P* < 0.001; **Figure 7F**). Next, the effects of coculture on the proliferation and migration of cancer cells were investigated. As demonstrated in **Figures 7G, H**, the data revealed that, compared to the control group, the cell viability and number of migrated cells were significantly promoted by silencing of CX3CR1 or coculture with THP-1 cells and were further elevated by simultaneous silencing of CX3CR1 and coculture with THP-1 cells (*P* < 0.05, *P* < 0.01, or *P* < 0.001). These data implied that CX3CR1 contributed to the recruitment and regulation of immune infiltrating cells and macrophage polarization in CRC, as well as TAM-induced CRC progression.

### Verification of CX3CR1 Expression in Human Colorectal Cancer Tissues

**Figure 8:**
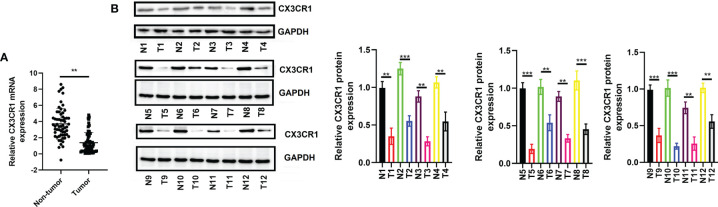
Expression of CX3CR1 in patients with CRC. **(A)** The mRNA expression of CX3CR1 in the enrolled 60 patients. **(B)** The protein expression of CX3CR1 in the 12 pairs of tissues. CX3CR1, C-X3-C motif chemokine receptor 1; CRC, colorectal cancer. **P < 0.01, ***P < 0.001 compared to the corresponding groups.

To further verify the expression of CX3CR1 and the potential functional role of CX3CR1 in CRC, we enrolled a total of 60 patients with CRC and analyzed the mRNA and protein expressions of CX3CR1 in the tumor tissues. As revealed in **Figure 8A**, the data showed that, compared to the non-tumor tissues, the mRNA expression of CX3CR1 was significantly downregulated in the tumor tissues (*P* < 0.01). In addition, 12 pairs of tissues were randomly chosen to assess the protein expression of CX3CR1. In line with the mRNA results, the protein expression of CX3CR1 was also statistically reduced in the tumor tissues compared with the non-tumor tissues (*P* < 0.01 or *P* < 0.001; **Figure 8B**). In addition, we investigated the relationship between CX3CR1 expression and CRC clinicopathological parameters, including gender, age, tumor location, TNM stage, and tumor size and differentiation. Among these 60 patients, 28 patients were categorized as high expression group and the remaining 32 patients were categorized as low expression group. As indicated in **Table 3**, CX3CR1 was not significantly related to age, gender, tumor location, size, and M stage but was associated with T and N stages, tumor differentiation, and prognosis of the tumor. These findings suggested that CX3CR1 may function as a potential prognostic biomarker in CRC.

**Table 3:** Correlation between CX3CR1 expression and clinicopathological characteristics of CRC.

Characteristics		Cases	CX3CR1 expression		*P* value
			Low	High	
Gender	Male	38	20	18	0.886
	Female	22	12	10	
Age	<60	26	14	12	0.944
	≥60	34	18	16	
Tumor location					
	Colon	40	21	19	0.582
	Rectum	20	12	8	
T stage					
	T1–2	36	12	24	0.000
	T3–4	24	18	4	
N stage					
	N0	32	12	20	0.009
	N1–2	28	20	8	
M stage					
	M0	34	16	18	0.265
	M1	22	16	10	
Differentiation					
	Low	12	5	7	0.003
	Medium	36	22	14	
	High	12	5	7	
Size					
	<4.5 cm	31	16	15	0.782
	≥4.5 cm	29	16	13	

CX3CR1, C-X3-C motif chemokine receptor 1; CRC, colorectal cancer.

## Discussion

CRC is a highly heterogeneous disease with increasing incidence and mortality. Immunotherapy has emerged as a novel approach for the management of CRC (19, 39, 40). Although many patients with CRC are immunoresponsive, some adverse effects, such as toxicity, have been reported recently (40). Therefore, there is an unmet need for exploring the targeting immunotherapy to the TME, since the TME plays an important role in the progression and development of cancers, as well as in responses to therapies, particularly immunotherapies (41). Moreover, the TME-related genes could be used as favorable predictors to evaluate patients’ survival, thereby improving the clinical consequence. In the current study, we performed a comprehensive analysis of the stromal and immune cells, the TME-associated genes, and the clinical prognosis of CRAD patients.

Firstly, we used the ESTIMATE algorithm to analyze the associations between the ImmuneScore, StromalScore, and EstimateScore and the stages and survival rates in CRAD patients acquired from TCGA database. ESTIMATE algorithm is a broad, novel, and reliable algorithm that has been administered in the data mining of many cancers (21). Our data showed that the StromalScore, ImmuneScore, and EstimateScore were generally decreased with the stage of disease malignancy. There were statistical differences in the latter two, which indicated that immune infiltration and tumor purity might significantly contribute to the development of CRAD. Moreover, the survival analysis revealed that the high three scores presented a longer lifetime than those with low scores. Combining these results, we demonstrated that the clinical consequences of CRAD patients were markedly affected by the TME, which were in line with previous studies (16, 42, 43).

Subsequently, we identified a total of 1,578 intersection genes. GO and KEGG pathway analyses established that the intersection genes were mainly enriched in the tumor immune response and the maintenance of TNM. For instance, the GO results indicated that the interaction genes were principally enriched in the regulation of leukocyte activation, T-cell activation, leukocyte migration/cell–cell adhesion/differentiation, and extracellular matrix (ECM)/structure organization, and the KEGG pathway demonstrated that they were specifically enriched in cytokine–cytokine receptor interaction and chemokine signaling pathway. Furthermore, according to the DEGs of the PPI network analysis, we identified and selected 62 hub genes as the first important module, and the KEGG pathway analyzed by ClueGO showed that these hub genes were also enriched in cytokine–cytokine receptor interaction and chemokine signaling pathway, etc. Our data supported previous investigations on the essential role of immune cells and ECM molecular components in the establishment of the TME, as well as the relationship between the progression and development of CRC and the TME (44–47).

To reveal the potential independent prognostic biomarkers for CRC, we performed univariate and multivariate Cox analysis by evaluating 62 hub genes and pathologic stages. After removal of insignificant variables, we found that pathologic stages (TNM) and several genes including GNG8, HRH3, CCL19, CXCR5, SSTR3, OPRL1, CX3CR1, and P2RY13 were significantly correlated with the prognosis of CRC in the univariate Cox analysis. At present, the TNM staging system has been well considered as the most frequently used predictor of OS and recurrence in CRC (48). Our results also confirmed that all stages were significantly correlated with the prognosis of CRC. GNG8 is a protein-coding gene, which is involved in GTPase activity and obsolete signal transducer activity. A previous bioinformatics analysis suggested that GNG8 was downregulated in CRC (49). However, the biological function of GNG8 in CRC remains uncertain to date. HRH3 is a presynaptic receptor, which mediates the discharge of histamine from histaminergic neurons and other neurotransmitters from different types of neurons (50). Recent research confirmed that HRH3 was involved in tumor growth and metastasis (51, 52). CCL19 belongs to the chemokine family, while CXCR5 and CX3CR1 are both chemokine receptors. These three factors play significant roles in many cancers, including CRC (53, 54). Interestingly, a previous study observed that CX3CR1 ectopic expression improved the recruitment of adoptively transferred T cells toward CX3CL1-generated cancers, leading to the augmentation of T-cell infiltration and reduction of tumor growth (55). SSTR3 is a well-known G-protein-coupled plasma membrane receptor and is activated by neuropeptides. It has been reported that SSTR3 was decreased with the Dukes’ stages in CRC (56). P2RY13 is a G-protein-coupled receptor, and it was reported to be decreased upon epidermal growth factor (EGF)- and hypoxia-induced epithelial–mesenchymal transition (EMT) in breast cancer cells (57, 58). Additionally, P2RY13 was also involved in the identification of M1 macrophages in CRC (59). However, multivariate Cox analysis showed that only T stage, N stage, and CX3CR1 were independent risk factors that could affect the prognosis of CRC. In addition, the survival of CX3CR1 also confirmed that the low score of CX3CR1 indicated a lower lifetime.

CX3CR1, located on chromosome 3p22.2, is a key chemokine receptor with a single ligand, which belongs to the G-protein-coupled receptor (GPCR) superfamily (60). It is a proinflammatory leukocyte receptor specific for the chemokine CX3CL1 [fractalkine (FKN)] (61). CX3CR1 includes four exons and three introns and is expressed by infiltrating immune cells (e.g., monocytes, CD8^+^ T cells, and NK cells) (62) and tissue-resident cells (e.g., macrophages and DCs) (63). Previous studies have revealed that the CX3CL1/CX3CR1 axis is responsible for numerous pathological processes, such as atherosclerosis (60), atherogenesis (64), nervous system diseases (65), vasculitis (66), abnormal heart function (67), and cancer development (68, 69). In addition, CX3CL1:CX3CR1 axis has been confirmed to play critical roles in the TME (70) and mediates several cellular functions, including cell proliferation, apoptosis, migration, and invasion by activation of phosphatidylinositol-3-kinases/protein-serine-threonine kinase (PI3K/AKT) and MAPK kinases, Src, and/or eNOS signaling pathways (71). However, the CX3CL1:CX3CR1 axis presents either pro- or antitumor effects in different cancers (72). For example, patients with a high expression of CX3CR1 were reported to be an independent negative prognosis factor in pancreatic ductal adenocarcinoma (73). Compared to the normal tissues, reduced expression of CX3CR1 was found in macrophages infiltrating CRC tissues (74). In contrast, a high expression of CX3CL1 was observed to have a positively strong association with a high number of stromal CD8^+^ T cells, NK cells, and intratumoral DCs in breast cancer (75). CX3CL1 was related to the density of TILs and was found to be one of the biomarkers for identifying CRC patients (76). Contrary to our results, a previous study suggested that CX3CR1 (lack of the allele I249) might play a limited or insignificant role in CRC, and plasma FKN/CX3CL1 does not appear to be a valuable tumor marker in CRC (77). These results implied that the effects of CX3CR1 might be heterogeneous even in the same cancers. To further confirm our bioinformatics results, we analyzed the expression of CX3CR1 in CRC tissues and cell lines, as well as the relationship between CX3CR1 and clinical parameters. As demonstrated in our *in vitro* experiments, we confirmed the lower expression of CX3CR1 in CRC tissues and cell lines. In addition, we observed that lower expression of CX3CR1 was correlated with tumor T and N stages, differentiation, and poorer prognosis.

Recently, the immune function of CX3CL1:CX3CR1 axis has been explored. For example, the expression of CX3CL1 has been confirmed to result in the infiltration of NK cells, DCs, CD4^+^, and CD8^+^ T cells into the tumor, which leads to an increase in the antitumor immune response (75). A previous research suggested that transduction with CX3CR1 increases T-cell recruitment into the TME in an animal model of CRC (55). On another front, CX3CR1^–^CD8^+^ T cells were reported to be functionally suppressed in the TME (78). To further explore the immune functions of CX3CR1, we investigated the associations between CX3CR1 expression and TILs and immune marker expression using TIMER database and CIBERSORT analysis. Interestingly, we observed that CX3CR1 expression was negatively related to purity but positively correlated with B cells, CD8^+^ T cells, CD4^+^ T cells, macrophages, neutrophils, and DCs. In addition, we observed that the high expression of CX3CR1 was positively correlated with resting DCs, resting mast cells, M2 macrophages, and plasma cells and negatively correlated with activated DCs, activated NK cells, and activated mast cells. These results indicated that CX3CR1 was associated with immune cell infiltration levels. The correlation between CX3CR1 and the expression of immune marker gene expression strongly suggested that CX3CR1 can regulate immune cell infiltration and interact within the TME. We detected a correlation between CX3CR1 and M1/M2 macrophage markers, which suggests that CX3CR1 might contribute to CRC by regulation of macrophage polarization. Macrophages are important innate immune cells that serve as a first line of defense against pathogenic insults to tissues. Nevertheless, TAM induces the expression of cytokines and chemokines that can inhibit antitumor immunity and promote cancer progression in different cancer types (79). Therefore, the protective effects of CX3CR1 on CRC might be by suppression of TAM-induced CRC progression. To confirm this, we used a coculture system to analyze the effects of coculture of THP-1 and CRC cells with low CX3CR1 expression on M1/M2 macrophage marker gene expression and cell proliferation and migration in CRC. As expected, coculture with THP-1-derived macrophages significantly promoted CRC cell proliferation and migration, which were in line with previous studies (80–82). Interestingly, our study found that simultaneous silencing of CX3CR1 and coculture with THP-1 cells further regulated macrophage polarization and promoted cell proliferation and migration of CRC cells. To our knowledge, this is the first report on the immune function of CX3CR1 with macrophages in cancer development.

Though our research achieved highly valued data, some limitations should be unneglectable. This study was performed only based on TCGA database; hence, a more comprehensive analysis should be implemented to illuminate the complicated relationship between the TME and CRC. Moreover, more immune-related experiments, such as the changes of CX3CR1 on the proportion changes of immune cells, should be performed to confirm the roles of CX3CR1 in the TME of CRC.

In conclusion, we comprehensively investigated the correlation between the TME-related genes and CRC by using the ESTIMATE algorithm based on TCGA database. Our data suggested that CX3CR1 might be a potential prognostic biomarker in the TME of CRC.

## Data Availability Statement

The original contributions presented in the study are included in the article/**Supplementary Material**. Further inquiries can be directed to the corresponding author.

## Ethics Statement

The studies involving human participants were reviewed and approved by The Medical Ethics Committee of Shengjing Hospital of China Medical University. The patients/participants provided their written informed consent to participate in this study.

## Author Contributions

Conception and design: ZS. Administrative support: QZ. Provision of study materials or patients: YY. Collection and assembly of data: YY and QZ. Data analysis and interpretation: YY and QZ. Article writing: All authors. Final approval of article: All authors. All authors contributed to the article and approved the submitted version.

## Funding

This research did not receive any specific grant from funding agencies in the public, commercial, or not-for-profit sectors.

## Conflict of Interest

The authors declare that the research was conducted in the absence of any commercial or financial relationships that could be construed as a potential conflict of interest.

## Publisher’s Note

All claims expressed in this article are solely those of the authors and do not necessarily represent those of their affiliated organizations, or those of the publisher, the editors and the reviewers. Any product that may be evaluated in this article, or claim that may be made by its manufacturer, is not guaranteed or endorsed by the publisher.
